# Hypertension, intracranial arteriosclerosis, and structural brain changes in patients with TIA or ischemic stroke

**DOI:** 10.1177/23969873241307099

**Published:** 2024-12-30

**Authors:** Xi Li, Bernhard P Berghout, Gijs van Rooijen, Mohammad Kamran Ikram, Bob Roozenbeek, Daniel Bos

**Affiliations:** 1 Department of Epidemiology, Erasmus University Medical Center, Rotterdam, The Netherlands; 2 Department of Public Health, Erasmus University Medical Center, Rotterdam, The Netherlands; 3 Department of Neurology, Erasmus University Medical Center, Rotterdam, The Netherlands; 4 Department of Radiology and Nuclear Medicine, Erasmus University Medical Center, Rotterdam, The Netherlands

**Keywords:** Ischemic stroke, hypertension, intracranial arteriosclerosis, mediation analysis, atrophy, white matter lesions, lacunes

## Abstract

**Introduction:**

Hypertension is a major risk factor of structural brain changes, including atrophy and cerebral small vessel disease. Intracranial arteriosclerosis could be an underlying mechanism between hypertension and structural brain changes. This study investigated whether intracranial carotid artery calcification (ICAC), as a proxy for intracranial arteriosclerosis, explains the association between hypertension and structural brain changes in patients with TIA or ischemic stroke.

**Patients and methods:**

About 968 patients (mean age 62.7 years) with TIA or ischemic stroke from a registry who underwent non-contrast CT (NCCT) and CT-angiography (CTA) were included in this study. Presence and volume (mm^3^) of ICAC were assessed on CTA. Subtypes of ICAC were assessed on NCCT, where ICAC was categorized into intimal and internal elastic lamina (IEL) type calcification. Structural brain changes, indicated by atrophy, periventricular and deep white matter lesions (WML), and lacunes were assessed on NCCT. Mediation analysis was performed using ICAC, ICAC volume, and ICAC subtypes as mediators.

**Results:**

ICAC was prevalent in 67.8% of patients, with 52.6% of them exhibiting intimal calcification, and 26.5% exhibiting IEL calcification. Atrophy, periventricular WML, deep WML, and lacunes were present in 48.1%, 56.4%, 43.0% and 17.1% of patients respectively. The presence of ICAC explained 7.1% of the association of hypertension with periventricular WML, 3.6% with deep WML, and 17.6% with lacunes. Hypertension was associated with increased atrophy through ICAC (OR: 1.02, 95% CI: 1.00–1.05). In subgroup analyses, IEL calcification partly explained the association between hypertension and periventricular WML (16.8%), and atrophy (OR: 1.12, 95% CI: 1.02–1.27). Intimal calcification did not explain any association.

**Conclusion:**

ICAC partially explained the association between hypertension and atrophy, periventricular and deep WML, and lacunes. Although intimal calcification was more prevalent in ischemic stroke patients, IEL calcification takes the leading role in explaining the association between hypertension and structural brain changes.

## Graphical abstract

**Graphical abstract:**
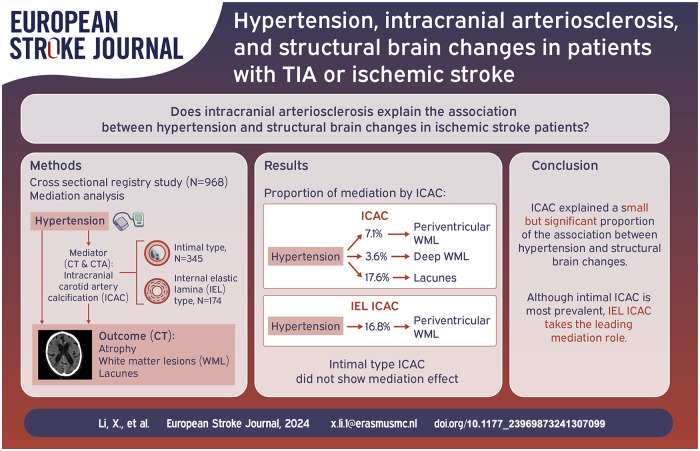

## Introduction

Structural brain changes, including brain atrophy, white matter lesions (WML), and lacunes are common in the stroke population, and contribute significantly to cognitive impairment.^1^ Within the complex etiological framework, hypertension is considered as a leading modifiable risk factor,^2^ yet the underlying pathophysiology remains unclear.

One explanation lies in arteriosclerosis of intracranial arteries, which is highly prevalent among ischemic stroke patients and is prominently associated with vascular brain disease.^3,4^ Intracranial carotid artery calcification (ICAC) is often used as a proxy indicator for intracranial arteriosclerosis,^5^ and has two distinct morphological subtypes: intimal calcification, considered as a marker of atherosclerotic plaque and related to luminal narrowing; and internal elastic lamina (IEL) calcification, thin and circular in shape, while related to arterial stiffening and reduced arterial compliance.^6^

Hypertension plays a pivotal role in the development of ICAC, with elevated blood pressure inducing remodeling of arterial wall structure and impairing endothelial function.^2^ Consequently, it can compromise cerebral blood flow autoregulation and causing brain tissue damage.^7^ Hence, ICAC may function as a mediator explaining the association between hypertension and structural brain changes. Prior research identified IEL calcification as the predominant ICAC subtype in the general population, highlighting it as the leading mechanism explaining the link between blood pressure and cerebral small vessel disease.^8^ In contrast, ischemic stroke patients may exhibit a higher prevalence of intimal calcification as it is considered as a major risk factor of cerebral ischemia.^3^ Despite the clinical importance, no prior study has explored the effects of ICAC subtypes on structural brain changes in ischemic stroke patients. This study aimed to investigate the magnitude of their contribution to the relation between hypertension and structural brain changes in patients with TIA and ischemic stroke, which may help to reveal the etiological pathway of hypertension on cognitive dysfunctioning relate to these structural brain changes.

## Methods

### Study population

1492 patients were selected from the Erasmus Stroke Study, a cross-sectional registry of patients with cerebrovascular diseases admitted to Erasmus MC, Rotterdam, The Netherlands from December 2005 to October 2010.^9^ Patients underwent were clinically evaluated in the outpatient clinic, emergency care department, or neurology ward. Blood samples, non-contrast CT (NCCT) and CT angiography (CTA) imaging were collected as standard care. Patients with a TIA or ischemic stroke event with available CT and CTA scans were included in this study. Exclusions applied if data on antihypertensive drug use and blood pressure were missing, or if CT and CTA results for the presence of ICAC were inconsistent, due to difficulty in detecting small calcification on CT scans with thick slices or distinguishing thin calcification from contrast material on CTA scans. This study followed the STROBE (Strengthening the Reporting of Observational Studies in Epidemiology) reporting guideline.^10^

### Image acquisition

Imaging was performed using a 16-slice, 64-slice, or 128-slice multidetector CT (MDCT) system (Brilliance 64, Philips Healthcare Systems, Eindhoven, Netherlands; Sensation 16, Sensation 64, Definition, Definition AS+ or Definition Flash, Siemens Medical Solutions, Erlangen, Germany) with a standardized optimized contrast-enhanced protocol. The scans ranged from the ascending aorta to the intracranial circulation (3 cm above the sella turcica). Detailed information on the scanning protocol is provided in the supplement. All MDCT and MDCTA scans were evaluated by trained readers blinded for clinical data.

### Assessment of intracranial carotid artery calcification

Calcification in the intracranial carotid artery was evaluated on axial CTA images, from the start of the petrous carotid canal until the circle of Willis. We manually delineated arterial calcification per slice. A threshold of 600 Hounsfield units (HU) on CTA scans was applied to differentiate arterial calcification from contrast material in the lumen.^11^ Calcification volume (mm^3^) was calculated by multiplying the number of pixels above 600 HU, pixel-size, and the slice increment. The total volume of ICAC was the sum of both left and right intracranial carotid arteries.

A previously validated method based on CT images was applied to differentiate ICAC subtypes,^6^ incorporating a composite score with weighting for calcification circularity, thickness, and continuity. The left and right arteries were categorized as predominantly intimal calcification (<7 points; i.e. thick, non-circular, and irregular), IEL calcification (⩾7 points; i.e. elongated, circular, and thin), or no calcification. The intra-rater reliability of scoring ICAC subtypes was evaluated on 30 randomly-selected scans, yielding Cohen kappa value of 0.850 for the right intracranial carotid artery, and 0.834 for the left. Patients were then classified into four subtype groups: intimal type ICAC (bilateral predominant intimal or combined with absent contralateral), IEL type ICAC (bilateral IEL or combined with absent contralateral), mixed type (predominant calcification on one side with a contrasting subtype on the other), and no ICAC (no calcifications).

### Assessment of structural brain changes

Structural brain changes indicated by atrophy, periventricular and deep WML, and lacunes were evaluated on NCCT scans. “Generalized Pasquier Scale,” a scale from 0 (not atrophic) to 3 (“knifeblade” atrophy), was used to visually assess the degree of general cerebral atrophy.^12^ The Fazekas et al.^13^ scale was applied to assess white matter changes with two 4-point (grade 0–3) scales for assessing periventricular and deep WML respectively. Lacunes were defined as 3–15 mm cerebrospinal fluid (CSF)-filled cavities in the sub-cortical regions, with the same density as CSF on NCCT.^14^

### Assessment of cardiovascular risk factors

Clinical information regarding cardiovascular risk factors and medication use were collected from hospital medical records at admission to the hospital. For patients with ischemic stroke, blood pressure was measured on day 2 to 5 of the hospital admission. For patients with TIA, blood pressure was measured during the diagnostic workup in the outpatient department. Hypertension was defined as the use of antihypertensive medication before the inclusion event to the hospital, or a systolic blood pressure ⩾140 mmHg and/or diastolic blood pressure ⩾90 mmHg.

Hypercholesterolemia was defined as the use of cholesterol lowering drugs before admission to the hospital or serum total cholesterol ⩾ 6.2 mmol/l. Diabetes mellitus was defined as the use of antidiabetic medication before the inclusion event, or fasting plasma glucose level ⩾ 7.0 mmol/l and/or a 2-h postload glucose level ⩾ 11.0 mmol/l. Smoking status was categorized into never or ever smoker. History of cardiovascular diseases, including previous TIA or ischemic stroke events, ischemic heart disease, atrial fibrillation, peripheral artery disease or other vascular diseases was collected.

### Statistical analysis

Patient characteristics are presented as means and standard deviations for normally distributed continuous data, medians with interquartile ranges for skewed variables, and percentages for categorical variables. For patients with ICAC, we log-transformed ICAC volume due to its right-skewed distribution.

**Figure 1.**
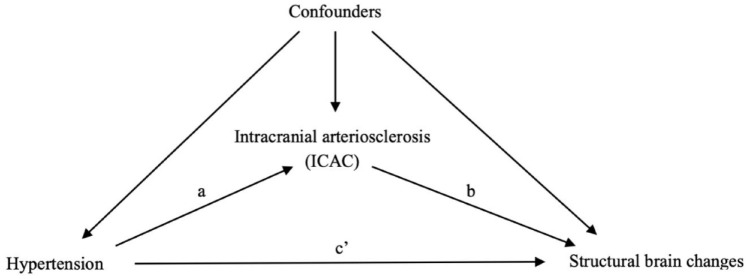
Directed acyclic graph for the association between hypertension, intracranial carotid arterial calcification (ICAC) and structural brain changes. Pathway a refers to the regression coefficient of the association between hypertension and ICAC; pathway b, the association between ICAC and structural brain changes, pathway c′, the association between hypertension and structural brain changes, controlling for ICAC. Confounders including age, sex, hypercholesterolemia, diabetes mellitus, smoking, and history of cardiovascular disease were adjusted in all analyses. Total effect (c) = direct effect (c′) + indirect effect (a × b). ICAC: intracranial carotid arterial calcification; IEL: internal elastic lamina.

To investigate how much of the association between hypertension and structural brain changes can be explained by the presence, the volume, and the subtypes of ICAC, we performed mediation analysis.^15,16^ As illustrated in Figure 1, this involved testing three pathways: step 1, the association between hypertension and each marker of structural brain changes (total effect); step 2, the association between hypertension and ICAC (pathway a); step 3, the association between ICAC and each marker of structural brain changes (pathway b).

We used logistic regression to analyze the association between hypertension and ICAC presence, linear regression for hypertension and log-transformed ICAC volume, and multinomial logistic regression for hypertension and ICAC subtypes (with no ICAC as the reference). Ordinal logistic regression model was used to analyze the association of hypertension and ICAC on brain atrophy and WML. Logistic regression was used to analyze the association with the presence of lacunes. All analyses were adjusted for covariates including hypercholesterolemia, diabetes mellitus, smoking, and history of cardiovascular diseases.

After estimating all pathways, mediation analysis was performed based on the regression-based approach,^17^ decomposing the total effect of hypertension on structural brain changes into indirect effect (i.e. mediated effect) and direct effect. Specifically, the indirect effect is the effect of hypertension on structural brain changes that can be explained by ICAC, and the direct effect is the effect that can be explained by any other factors rather than ICAC. The proportion of mediation was calculated by dividing the indirect effect by the total effect.

All analyses were conducted in the entire sample, and in two subgroups: patients with no ICAC plus those with intimal type ICAC; patients with no ICAC plus those with IEL type ICAC. Mediation analyses were performed in these two subgroups separately to compare the mediated effect of intimal and IEL type calcification. Covariates were selected based on the association with hypertension, ICAC, and structural brain changes and adjusted for in all models.

Statistical analyses were conducted using R statistical software 4.3.2 (mice 3.16.0, MASS 7.3.60, CMAverse 0.1.0 R packages). Missing values in the covariates (i.e. total cholesterol level and smoking) were fewer than 10% and were imputed using 10-fold multiple imputation with 10 iterations. Blood pressure values were missing for 104 patients (10.7%), and these were imputed under the assumption of missing at random. A sensitivity analysis excluding patients with imputed blood pressure values was conducted to assess the impact of imputation on the results.

To address the variation in CT scanner type regarding the detection and classification of ICAC, we conducted a sensitivity analysis adjusting for scanner type in the association between ICAC and structural brain changes.

## Results

### Study population

**Figure 2.**
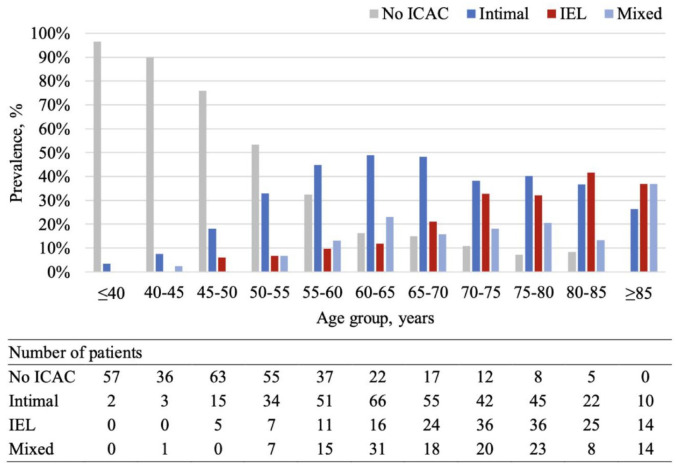
Prevalence of morphological subtypes of ICAC across every 5-year age group. The figure demonstrates the prevalence of each subtype of ICAC when stratified by every 5-year age group. As the age increases, the prevalence of having no ICAC decreases, whereas the prevalence of IEL type ICAC increases. Intimal type ICAC remained to be the most prevalent subtype between the age of 55–80. ICAC: intracranial carotid arterial calcification; IEL: internal elastic lamina.

968 patients were included in the analysis (Supplemental Figure S1). Excluded patients were significantly older, more with hypertension and history of cardiovascular disease, and more present with ischemic stroke instead of TIA (Supplemental Table S1). Table 1 summarizes the included patient characteristics. The mean age of the patients included in the study was 62.7 ± 14.1 years old and 45.6% of them were female. Among patients with ICAC (*n* = 656; 67.8%), 345 (52.6%) patients predominantly showed intimal calcification, 174 (26.5%) with IEL calcification, and 137 (20.9%) with mixed calcification. Intimal calcification was the most prevalent subtype from age 55 to 80 in the stroke population. The prevalence of IEL calcification keeps increasing with the increase of age (Figure 2). Atrophy, periventricular WML, deep WML, and lacunes were present in 48.1%, 56.4%, 43.0%, and 17.1% of patients respectively.

**Table 1.** Characteristics of study participants.

Patient characteristics	All participants (*n* = 968)
Age, years	62.7 (14.1)
Women	441 (45.6)
Systolic blood pressure, mmHg	129.8 (17.7)
Diastolic blood pressure, mmHg	75.5 (9.9)
Antihypertensive drug use	492 (50.8)
Hypertension	603 (62.3)
Hypercholesterolemia	434 (44.8)
Diabetes Mellitus	295 (30.5)
Smoking	638 (65.9)
History of cardiovascular disease	460 (47.5)
NIHSS score	1.0 (0.0–4.0)
Symptom duration < 24 h (TIA)	386 (39.9)
TOAST classification	
Large artery atherosclerosis	183 (18.9)
Cardioembolism	122 (12.6)
Small vessel disease	186 (19.2)
Other determined etiology	54 (5.6)
Undetermined etiology	423 (43.7)
Intracranial carotid artery calcification	
ICAC	656 (67.8)
Intimal type calcification	345 (52.6)
IEL type calcification	174 (26.5)
Mixed type calcification	137 (20.9)
ICAC volume, mm^3^	23.1 (5.1–69.5)
Structural brain changes	
Atrophy	466 (48.1)
Periventricular white matter lesions	546 (56.4)
Deep white matter lesions	416 (43.0)
Lacunes	166 (17.1)

Continuous variables were presented as mean (±SD) or median (interquartile range). Categorical variables were shown as numbers of patients and frequencies (%).

ICAC: intracranial carotid artery calcification; IEL: internal elastic lamina.

### Association between hypertension, ICAC, and structural brain changes

Hypertension was associated with a higher prevalence of ICAC (adjusted Odds Ratio (aOR): 1.63, 95% CI: 1.11–2.38), in particular with IEL calcification (aOR: 2.49, 95% CI: 1.45–4.28) (Table 2). Similarly, hypertension was associated with more severe periventricular WML (adjusted common Odds Ratio (acOR): 1.50, 95% CI: 1.13–2.00) and deep WML (acOR: 1.82, 95% CI: 1.34–2.47) (Table 3). The presence of ICAC was independently associated with more severe atrophy (acOR: 2.15, 95% CI: 1.44–3.20), periventricular WML (acOR: 1.84, 95% CI: 1.30–2.62), deep WML (acOR: 1.49, 95% CI 1.02–2.18), and the presence of lacunes (aOR: 1.70, 95% CI: 1.01–2.88). With absent ICAC being the reference group, IEL calcification has a more pronounced association with atrophy (acOR: 2.67, 95% CI 1.62–4.40), periventricular WML (acOR: 1.93, 95% CI 1.24–3.01) and deep WML (acOR: 1.69, 95% CI 1.06–2.70) than intimal calcification (Table 3). For lacunes, the only significant association was with mixed calcification (aOR: 2.18, 95% CI 1.15–4.13).

**Table 2.** Association of hypertension with the presence and subtypes of intracranial carotid arterial calcification (ICAC).

All patients (*n* = 968)		Hypertension	
		OR	95% CI
ICAC presence versus absence		1.63	1.11-2.38
ICAC subtypes	Intimal ICAC versus no ICAC	1.41	0.95-2.10
	IEL ICAC versus no ICAC	2.49	1.45–4.28
	Mixed ICAC versus no ICAC	2.01	1.18–3.42

Binary logistic regression was performed for the presence of ICAC. Multinomial logistic regression was performed for ICAC subtypes. Models were adjusted for age, sex, hypercholesterolemia, diabetes, smoking and history of cardiovascular diseases.

ICAC: intracranial carotid arterial calcification; IEL: internal elastic lamina.

**Table 3.** Association of hypertension and intracranial carotid arterial calcification (ICAC) with structural brain changes.

Exposure	Outcome							
	Atrophy		Periventricular WML		Deep WML		Lacunes	
	OR	95% CI	OR	95% CI	OR	95% CI	OR	95% CI
All patients (*n* = 968)								
Hypertension	0.81	0.59–1.10	1.50	1.13–2.00	1.82	1.34–2.47	1.24	0.83–1.87
Presence of ICAC	2.15	1.44–3.20	1.84	1.30–2.62	1.49	1.02–2.18	1.70	1.01–2.88
ICAC subtypes								
Intimal ICAC vs no ICAC	1.87	1.23–2.83	1.76	1.22–2.54	1.34	0.90–2.00	1.60	0.93–2.77
IEL ICAC vs no ICAC	2.67	1.62–4.40	1.93	1.24–3.01	1.69	1.06–2.70	1.61	0.84–3.06
Mixed ICAC vs no ICAC	2.83	1.71–4.68	2.07	1.32–3.25	1.82	1.13–2.93	2.18	1.15–4.13

Ordinal logistic regression was performed for brain atrophy, periventricular WML, and deep WML. Binary logistic regression was performed for the presence of lacunes. Models were adjusted for age, sex, hypercholesterolemia, diabetes, smoking and history of cardiovascular diseases. The model for the association between ICAC, ICAC subtypes, and structural brain changes was adjusted for hypertension additionally.

ICAC: intracranial carotid arterial calcification; IEL: internal elastic lamina; WML: white matter lesions.

### ICAC as a mediator between hypertension and structural brain changes

In the mediation analyses, the presence of ICAC explained 7.1% of the association of hypertension with periventricular WML, 3.6% of the association with deep WML, and 17.6% with the presence of lacunes (Table 4). Hypertension was associated with more severe brain atrophy through the mediation of ICAC (acOR: 1.02, 95% CI: 1.00–1.05), while the direct effect of hypertension on brain atrophy was toward the opposite direction (acOR: 0.82, 95% CI 0.64–1.04).

**Table 4.** Estimated mediation effect of ICAC, log-ICAC volume, and ICAC subtypes in the association between hypertension and structural brain changes.

Exposure	Mediator	Outcome	OR (95% CI)			Proportion of mediation	*p*-Value of the mediated effect
			Total effect	Direct effect	Indirect effect		
Hypertension	ICAC	Atrophy	0.820 (0.635–1.041)	0.802 (0.623–1.02)	1.023 (1.001–1.045)	N/A^a^	0.036
		PVWML	1.338 (1.053–1.686)	1.314 (1.036–1.651)	1.018 (1.001–1.039)	7.1%	0.044
		DWML	1.664 (1.280–2.210)	1.641 (1.267–2.174)	1.015 (0.999–1.034)	3.6%	0.074
		Lacunes	1.223 (0.835–1.804)	1.183 (0.816–1.729)	1.033 (1.000–1.076)	17.6%	0.052
	Intimal ICAC	Atrophy	0.919 (0.681–1.286)	0.918 (0.667–1.255)	1.001 (0.987–1.059)	N/A	0.274
		PVWML	1.468 (1.116–2.043)	1.467 (1.100–2.022)	1.001 (0.988–1.052)	0.2%	0.306
		DWML	1.916 (1.411–2.831)	1.915 (1.400–2.842)	1.001 (0.988–1.037)	0.1%	0.558
		Lacunes	1.177 (0.761–2.040)	1.177 (0.748–2.010)	1.000 (0.987–1.069)	0.0%	0.334
	IEL ICAC	Atrophy	0.781 (0.528–1.179)	0.699 (0.480–1.056)	1.118 (1.017–1.268)	N/A	0.018
		PVWML	1.654 (1.196–2.290)	1.544 (1.125–2.167)	1.074 (1.004–1.158)	16.8%	0.026
		DWML	1.812 (1.234–2.799)	1.722 (1.166–2.613)	1.052 (0.991–1.129)	11.0%	0.132
		Lacunes	1.744 (0.936–3.731)	1.635 (0.861–3.392)	1.067 (0.991–1.209)	14.7%	0.120
	Log ICAC volume (per SD increase)	Atrophy	0.806 (0.610–1.050)	0.809 (0.615–1.055)	0.997 (0.973–1.021)	1.4%	0.836
		PVWML	1.122 (0.881–1.421)	1.126 (0.885–1.431)	0.996 (0.963–1.024)	N/A	0.848
		DWML	1.415 (1.074–1.877)	1.420 (1.083–1.879)	0.996 (0.959–1.027)	N/A	0.846
		Lacunes	1.024 (0.682–1.638)	1.029 (0.680–1.674)	0.995 (0.959–1.037)	N/A	0.850

Mediation analysis was performed in all patients and in two subgroups of patients with intimal type or IEL type ICAC. All models were adjusted for age, sex, hypercholesterolemia, diabetes, smoking and history of cardiovascular diseases.

a N/A indicates not applicable, proportion of mediation was not calculated when the direct and indirect effect operate in the opposite directions.

ICAC: intracranial carotid artery calcification; IEL: internal elastic lamina; PVWML: periventricular white matter lesions; DWML: deep white matter lesions.

For mediation analyses performed in the two subgroups of patients with no ICAC + patients with intimal type ICAC (*n* = 657) and patients with no ICAC + patients with IEL type ICAC (*n* = 486), IEL calcification explained 16.8% of the association between hypertension and periventricular WML. The mediated effect of IEL calcification on brain atrophy was significant (acOR: 1.12, 95% CI: 1.02–1.27) but on the opposite direction of the direct effect of hypertension on brain atrophy (acOR: 0.78, 95% CI: 0.53–1.18). No significant mediated effect of intimal calcification was observed. We also tested the interaction between hypertension and ICAC, but we didn’t find significant interaction effect, thus interaction terms were left out in further mediation analyses.

In the sensitivity analysis, we excluded 104 patients with missing values on blood pressure, and ended up with 864 patients for the mediation analysis. The mediation effects found were similar (Supplemental Table S4). The association between ICAC and structural brain changes remains consistent after adjusting for CT scanner type (Supplemental Table S5).

## Discussion

In this cross-sectional study of patients with TIA or ischemic stroke, the presence of ICAC was observed in two-thirds of the patients. Our mediation analyses results revealed that the presence of ICAC partially explained the effect of hypertension on brain atrophy, periventricular WML, deep WML, and lacunes. Despite the higher prevalence of intimal calcification in stroke patients, the mediated effect was mainly driven by IEL calcification.

### Prevalence of intimal and IEL calcification in patients with ischemic stroke or TIA

Intimal calcification was found to be the most prevalent type of intracranial arteriosclerosis in patients with TIA or ischemic stroke, which is consistent with the recognized involvement of atherosclerosis in the pathophysiology of cerebral ischemia.^3^ However, a previous study reported IEL calcification to be the most prevalent subtype,^18^ which could be explained by the relatively older age of their study participants compared to this study, as IEL calcification is strongly associated with older age. Stratifying the prevalence by every 5-year age group revealed an increasing predominance of the IEL subtype with advancing age, which aligns with previous research.^19^ These findings highlight that calcification along the IEL of intracranial arteries is an age-related process.

### Association between hypertension, ICAC, and structural brain changes

The effect of hypertension and ICAC varies between different markers of structural brain changes. The opposite direction of direct and indirect effect on brain atrophy indicates that while higher blood pressure may serve as a compensatory mechanism for inadequate cerebral blood flow,^20,21^ the presence of ICAC counteracts with this protective effect, especially for IEL calcification. For lacunes, although no significant direct effect of hypertension was found, the mediation through ICAC was significant. This is consistent with the pathophysiological perspective, where hypertension contributes to lacune formation by inducing changes in small arteries, and increasing their susceptibility to occlusion.^22^ Compared to atrophy and lacunes, the direct effect of hypertension on WML was substantially larger and remained to be significant. This implies that while hypertension is a strong risk factor of WML, the effect primarily operates through other pathways than ICAC, such as inflammation and oxidative stress,^2^ and increased blood-brain barrier permeability.^23^

### Mediated effect through intimal and IEL calcification

The mediation analyses revealed a small but significant contribution of ICAC to the effect of hypertension on structural brain changes, with IEL calcification accounting for a larger proportion of the mediated effect compared to intimal type. This was expected as more pronounced association was observed between hypertension and IEL calcification (pathway a), and between IEL calcification and structural brain changes (pathway b). A larger mediated effect of IEL calcification was also observed in a prior study conducted in the general population (age 68.0 ± 5.7 years) where IEL calcification was the most common form of ICAC.^8^ This implies that the prevalence of ICAC subtypes may change with age and according to whether people suffered from stroke, but IEL calcification remains the leading mechanism explaining the association between hypertension and structural brain changes. The potential mechanism involves IEL calcification as an indicator of arterial aging and stiffness, contributing to impaired cerebral autoregulation and the progression of neurodegenerative processes by facilitating excessive pulsatile pressure in the cerebral microcirculation.

### Strengths and limitations

This study used mediation analysis to explain the underlying mechanism of how hypertension is related to structural brain changes in patients with TIA or ischemic stroke, where the prevalence of ICAC and structural brain changes are different compared to the general population.

Several limitations require discussion. First, with the cross-sectional study design, we cannot draw a causal conclusion on the effect of hypertension on ICAC. Nevertheless, the biological plausibility of hypertension leading to arteriosclerosis and subsequent brain tissue damage supports the temporal ordering of variables. Future longitudinal studies incorporating detailed blood pressure measurements over time would provide more valuable insights. Second, valid estimates of mediation effect assume that baseline covariates fully control for exposure-mediator, mediator-outcome, exposure-outcome confounding. However, there could still be residual confounding that we could not adjust for, leading to our results being biased upwards. On the other hand, given that the excluded patients were significantly older, and with more hypertension, the exclusion likely led to an underestimation of the observed associations. Third, the CT scans we used were 3–4.8 mm thick, increasing the possibility of missing small calcifications or misclassifying subtypes due to the difficulty of assessing continuity with thick slices. CT measurements of white matter lesions (WML) may have lower sensitivity; however, prior studies have shown substantial agreement between CT and MRI in WML visual rating scales, supporting CT as a reliable tool for WML assessment in acute stroke events.^24^ Lastly, the current mediation R package does not support mediation analysis with multinomial mediator. Therefore, we chose to compare the mediation effect of two ICAC subtypes in two subgroups of patients. However, it aligns with our research aim of comparing the mediating roles of two ICAC subtypes. The more pronounced association observed between IEL calcification and hypertension, along with structural brain changes also supports our finding of the significant mediated effect of IEL calcification.

## Conclusion

This study showed that ICAC partially explained the association between hypertension and brain atrophy, periventricular and deep WML, and lacunes. Although a higher prevalence of intimal calcification was found in patients with ischemic stroke or TIA, the mediated effect was mainly driven by IEL type calcification.

## Supplemental Material

sj-docx-1-eso-10.1177_23969873241307099 – Supplemental material for Hypertension, intracranial arteriosclerosis, and structural brain changes in patients with TIA or ischemic stroke Supplemental material, sj-docx-1-eso-10.1177_23969873241307099 for Hypertension, intracranial arteriosclerosis, and structural brain changes in patients with TIA or ischemic stroke by Xi Li, Bernhard P Berghout, Gijs van Rooijen, Mohammad Kamran Ikram, Bob Roozenbeek and Daniel Bos in European Stroke Journal
