# Supplementary material for: Hypertension, intracranial arteriosclerosis, and structural brain changes in patients with TIA or ischemic stroke
Source: Eur Stroke J. 2024 Dec 30;10(3):804–12. doi: 10.1177/23969873241307099 (PMC11683788; doi:10.1177/23969873241307099)
Supplement: sj-docx-1-eso-10.1177_23969873241307099 – Supplemental material for Hypertension, intracranial arteriosclerosis, and structural brain changes in patients with TIA or ischemic stroke [file sj-docx-1-eso-10.1177_23969873241307099.docx]

**Supplementary materials**

**Scan protocol**

**Figure S1**

**Table S1-S3**

Scan protocol

MDCT and MDCTA Data Acquisition

Image acquisition was performed using a 16-slice, 64-slice, or 128-slice MDCT system (Sensation 16, Sensation 64, Definition, Definition AS+ or Definition Flash, Siemens Medical Solutions, Erlangen, Germany) using a standardized optimized contrast-enhanced protocol (120 kVp; 180–200 reference mAs; collimation 16×0.75 mm, 32×2×0.6 mm, or 64×2×0.6 mm; pitch <1).

The MDCTA scan ranged from the ascending aorta to the intracranial circulation (3 cm above the sella turcica). All patients received 80 mL of contrast agent (320 mg/mL iodixanol, Visipaque, Amersham Health, Little Chalfont, United Kingdom), followed by 45-mL saline bolus chaser, both at an injection rate of 4 to 5 mL/s. Real-time bolus tracking at the level of the ascending aorta was used to synchronize passage of contrast agent and data acquisition. Image reconstructions were made with field of view of 120 mm, matrix size 512×512, slice thickness 1.0 or 0.75 mm, increment 0.6 to 0.4 mm, and an intermediate reconstruction algorithm.

The MDCT brain scan ranged from the foramen magnum to the vertex. Image reconstructions were made with a 200 to 250 mm field of view, matrix size 512×512, slice thickness 3 to 5 mm, and an intermediate reconstruction algorithm.

Patients with TIA or ischemic stroke from Erasmus Stroke Study

N = 1492

Excluded from analysis:

CTA scans not available or not evaluable due to image artefacts

n = 247

Patients with available CTA scans

N = 1245

Excluded from analysis:

CT scans not available or not evaluable due to image artefacts

n = 124

Patients with available CT scans

N = 1121

Excluded from analysis:

Patients with inconsistent results on the presence of ICAC on CT and CTA scans

n = 150

Patients with available data on blood pressure and antihypertensive drug use

N = 971

Excluded from analysis:

Patients with missing values on both antihypertensive drug use and blood pressure values

n = 3

Patients included in the analysis

N = 968

Figure S1 Flowchart for study participants

Abbreviations: ICAC, intracranial carotid arterial calcification.

Table S1

Baseline characteristics of included and excluded patients

| Characteristic | Included patients  N=968 | Excluded patients  N=524 | p value |
| --- | --- | --- | --- |
| Age, years | 62.6 (14.2) | 64.9 (15) | 0.004 |
| Women | 441/968 (45.6) | 274/524 (52.3) | 0.015 |
| Systolic blood pressure, mmHg | 129.6 (18.4) | 132.1 (20.6) | 0.032 |
| Diastolic blood pressure, mmHg | 75.6 (10.4) | 75.9 (12.1) | 0.640 |
| Antihypertensive drug use | 491/967 (50.8) | 319/521 (61.2) | <0.001 |
| Hypertension | 603/968 (62.3) | 376/524 (71.8) | <0.001 |
| Hypercholesterolemia | 433/934 (46.4) | 240/481 (49.9) | 0.228 |
| Diabetes Mellitus | 295/968 (30.5) | 173/524 (33.0) | 0.342 |
| Smoking | 572/891 (64.2) | 269/431 (62.4) | 0.568 |
| History of cardiovascular disease | 458/963 (47.6) | 290/519 (55.9) | 0.003 |
| NIHSS score | 1.0 (0.0-4.0) | 2.0 (0.0-7.0) | <0.001 |
| Symptom duration < 24h (TIA) | 383/963 (39.8) | 153/522 (29.3) | <0.001 |

Notes: Continuous variables were presented as mean (±SD) or median (interquartile range). Categorical variables were shown as numbers of patients and frequencies (%).

Table S2 Association of hypertension and intracranial carotid arterial calcification (ICAC in subgroups of patients with different ICAC subtypes

|  |  | Hypertension | |
| --- | --- | --- | --- |
|  |  | OR | 95% CI |
| Participants with no ICAC + participants with intimal ICAC (n=657) | | |  |
|  | Intimal ICAC vs no ICAC | 1.41 | (0.95-2.10) |
| Participants with no ICAC + participants with IEL ICAC (n=486) | |  |  |
|  | IEL ICAC vs no ICAC | 2.49 | (1.45-4.28) |
| Participants with no ICAC + participants with mixed ICAC (n=449) | | |  |
|  | Mixed ICAC vs no ICAC | 2.01 | (1.18-3.42) |

Notes: Binary logistic regression was performed in each subgroup. Models were adjusted for age, sex, hypercholesterolemia, diabetes, smoking and history of cardiovascular diseases.

Abbreviations: ICAC, intracranial carotid arterial calcification; IEL, internal elastic lamina.

Table S3 Association of hypertension and intracranial carotid arterial calcification (ICAC with structural brain changes in subgroups of patients with different ICAC subtypes

|  | Atrophy | | | PVWML | | | DWML | | | | | Lacunes | | | |
| --- | --- | --- | --- | --- | --- | --- | --- | --- | --- | --- | --- | --- | --- | --- | --- |
|  | OR | 95% CI | OR | | 95% CI | | OR | 95% CI | | | OR | | 95% CI | |  |
| Patients with no ICAC + patients with intimal ICAC (n=657) | | | | | | | |  |  |  | | |  |  | |
| Hypertension | 0.94 | (0.64-1.37) | 1.64 | | (1.16-2.31) | | 2.06 | (1.41-3.02) | | | 1.21 | | (0.73-2.01) | |  |
| Intimal ICAC vs no ICAC | 1.85 | (1.20-2.83) | 1.65 | | (1.13-2.41) | | 1.20 | (0.79-1.83) | | | 1.51 | | (0.86-2.67) | |  |
| Patients with no ICAC + patients with IEL ICAC (n=486) | | | | | | |  | | |  | | |  |  | |
| Hypertension | 0.78 | (0.49-1.25) | 1.94 | | | (1.26-2.99) | 2.05 | (1.26-3.31) | | | 1.78 | | (0.92-3.43) | | |
| IEL ICAC vs no ICAC | 3.97 | (2.24-7.04) | 2.04 | | | (1.22-3.39) | 1.63 | (0.95-2.78) | | | 1.80 | | (0.86-3.79) | | |
| Patients with no ICAC + patients with mixed ICAC (n=449) | | | | | | |  | | |  | | |  |  | |
| Hypertension | 0.82 | (0.50-1.34) | 2.13 | | | (1.35-3.34) | 2.40 | (1.45-3.97) | | | 1.67 | | (0.87-3.22) | | |
| Mixed ICAC vs no ICAC | 3.66 | (2.09-6.40) | 2.14 | | | (1.29-3.55) | 1.81 | (1.07-3.07) | | | 2.30 | | (1.12-4.72) | | |

Notes: Ordinal logistic regression was performed for brain atrophy, periventricular WML, and deep WML. Binary logistic regression was performed for the presence of lacunes. Models were adjusted for age, sex, hypercholesterolemia, diabetes, smoking and history of cardiovascular diseases. The model for the association between ICAC and structural brain changes was adjusted for hypertension additionally

Abbreviations: ICAC, intracranial carotid artery calcification; IEL, internal elastic lamina; PVWML, periventricular white matter lesions; DVWML, deep white matter lesions.

Table S4 Sensitivity analysis of estimated mediation effect of ICAC, log-ICAC volume, and ICAC subtypes in the association between hypertension and structural brain changes

| Exposure | Mediator | Outcome | OR (95% CI) | | | Proportion of mediation | P-value of the mediated effect |
| --- | --- | --- | --- | --- | --- | --- | --- |
|  |  |  | Total effect | Direct effect | Indirect effect |  |  |
| Hypertension | ICAC | Atrophy | 0.85 (0.65, 1.11) | 0.82 (0.63, 1.10) | 1.03 (1.00, 1.05) | N/A* | 0.060 |
|  |  | PVWML | 1.51 (1.18, 1.96) | 1.47 (1.16, 1.93) | 1.03 (1.00, 1.04) | 8% | 0.072 |
|  |  | DWML | 2.01 (1.45, 2.85) | 1.96 (1.43, 2.80) | 1.02 (1.00, 1.05) | 4% | 0.122 |
|  |  | Lacunes | 1.15 (0.75, 1.81) | 1.11 (0.73, 1.76) | 1.03 (1.00, 1.09) | 23% | 0.074 |
|  |  |  |  |  |  |  |  |
|  | log ICAC volume  (per SD increase) | Atrophy | 0.84 (0.63, 1.14) | 0.85 (0.63, 1.14) | 0.99 (0.96, 1.02) | 4% | 0.492 |
|  |  | PVWML | 1.24 (0.92, 1.65) | 1.25 (0.94, 1.66) | 0.99 (0.96, 1.02) | N/A | 0.488 |
|  |  | DWML | 1.66 (1.19, 2.33) | 1.68 (1.21, 2.34) | 0.99 (0.94, 1.03) | N/A | 0.482 |
|  |  | Lacunes | 0.92 (0.58, 1.48) | 0.93 (0.59, 1.49) | 0.99 (0.93, 1.03) | 15% | 0.520 |
|  |  |  |  |  |  |  |  |
|  | Intimal type ICAC | Atrophy | 1.00 (0.70, 1.43) | 0.96 (0.69, 1.39) | 1.04 (0.99, 1.07) | N/A | 0.242 |
|  |  | PVWML | 1.67 (1.18, 2.38) | 1.62 (1.16, 2.33) | 1.03 (0.99, 1.06) | 7% | 0.268 |
|  |  | DWML | 2.30 (1.54, 3.42) | 2.29 (1.53, 3.37) | 1.01 (0.98, 1.04) | 1% | 0.592 |
|  |  | Lacunes | 1.21 (0.73, 2.15) | 1.19 (0.72, 2.12) | 1.01 (0.98, 1.08) | 8% | 0.284 |
|  |  |  |  |  |  |  |  |
|  | IEL type ICAC | Atrophy | 0.78 (0.52, 1.21) | 0.68 (0.46, 1.04) | 1.15 (1.02, 1.33) | N/A | 0.012 |
|  |  | PVWML | 1.85 (1.30, 2.81) | 1.74 (1.21, 2.62) | 1.07 (1.00, 1.18) | 13% | 0.042 |
|  |  | DWML | 2.18 (1.42, 3.74) | 2.07 (1.32, 3.49) | 1.06 (1.00, 1.18) | 10% | 0.076 |
|  |  | Lacunes | 1.56 (0.79, 3.61) | 1.48 (0.72, 3.35) | 1.05 (0.98, 1.23) | 14% | 0.140 |

Notes: Sensitivity analysis was conducted where patients with imputed blood pressure values were excluded, and ended up with 864 patients. Mediation analysis was performed in all patients and in two subgroups of patients with intimal type or IEL type ICAC. All models were adjusted for age, sex, hypercholesterolemia, diabetes, smoking and history of cardiovascular diseases. *N/A indicates not applicable, proportion of mediation was not calculated when the direct and indirect effect operate in the opposite directions. Abbreviations: ICAC, intracranial carotid artery calcification; IEL, internal elastic lamina; PVWML, periventricular white matter lesions; DWML, deep white matter lesions.

Table S5

Association between ICAC presence, ICAC subtypes with structural brain changes with and without adjusting for scanner types

|  | Atrophy | | | | Lacunes | | | |
| --- | --- | --- | --- | --- | --- | --- | --- | --- |
|  | Unadjusted | | Adjusted | | Unadjusted | | Adjusted | |
|  | OR | 95% CI | OR | 95% CI | OR | 95% CI | OR | 95% CI |
| Presence of ICAC | 2.15 | 1.44-3.20 | 2.18 | 1.46-3.24 | 1.70 | 1.01-2.88 | 1.72 | 1.02-2.91 |
| ICAC subtypes |  |  |  |  |  |  |  |  |
| Intimal vs no ICAC | 1.87 | 1.23-2.83 | 1.90 | 1.25-2.88 | 1.60 | 0.93-2.77 | 1.62 | 0.94-2.80 |
| IEL vs no ICAC | 2.67 | 1.62-4.40 | 2.67 | 1.62-4.39 | 1.61 | 0.84-3.06 | 1.61 | 0.84-3.06 |
| Mixed vs no ICAC | 2.83 | 1.71-4.68 | 2.85 | 1.72-4.72 | 2.18 | 1.15-4.13 | 2.21 | 1.16-4.19 |
|  |  |  |  |  |  |  |  |  |
|  | Periventricular WML | | | | Deep WML | | | |
|  | Unadjusted | | Adjusted | | Unadjusted | | Adjusted | |
|  | OR | 95% CI | OR | 95% CI | OR | 95% CI | OR | 95% CI |
| Presence of ICAC | 1.84 | 1.30-2.62 | 1.84 | 1.30-2.62 | 1.49 | 1.02-2.18 | 1.51 | 1.03-2.21 |
| ICAC subtypes |  |  |  |  |  |  |  |  |
| Intimal vs no ICAC | 1.76 | 1.22-2.54 | 1.76 | 1.22-2.53 | 1.34 | 0.90-2.00 | 1.35 | 0.91-2.02 |
| IEL vs no ICAC | 1.93 | 1.24-3.01 | 1.94 | 1.24-3.02 | 1.69 | 1.06-2.70 | 1.70 | 1.06-2.71 |
| Mixed vs no ICAC | 2.07 | 1.32-3.25 | 2.07 | 1.32-3.25 | 1.82 | 1.13-2.93 | 1.84 | 1.14-2.96 |

Notes: Binary logistic regression was performed for the presence of ICAC. Multinomial logistic regression was performed for ICAC subtypes. Models were all adjusted for age, sex, hypercholesterolemia, diabetes, smoking and history of cardiovascular diseases.
